# Analysis of self-report and biochemically verified tobacco abstinence outcomes with missing data: a sensitivity analysis using two-stage imputation

**DOI:** 10.1186/s12874-018-0635-2

**Published:** 2018-12-18

**Authors:** Yiwen Zhang, Xianghua Luo, Chap T. Le, Jasjit S. Ahluwalia, Janet L. Thomas

**Affiliations:** 1 Joseph J. Zilber School of Public Health, University of Wisconsin-Milwaukee, 1240 N 10th St, Milwaukee, WI 53205 USA; 2 School of Public Health, Division of Biostatistics, University of Minnesota, 420 Delaware St. SE, MMC 303, Minneapolis, MN 55455 USA; 3 University of Minnesota Masonic Cancer Center, Minneapolis, MN 55455 USA; 4 Brown University School of Public Health, Box G-S121-5, Providence, RI 02912 USA; 5 Division of General Internal Medicine, Department of Medicine, University of Minnesota, 717 Delaware St. SE, Minneapolis, MN 55414 USA

**Keywords:** Abstinence outcome, Imputation, Missing data, Sensitivity analysis

## Abstract

**Background:**

Missing data are common in tobacco studies. It is well known that from the observed data alone, it is impossible to distinguish between missing mechanisms such as missing at random (MAR) and missing not at random (MNAR). In this paper, we propose a sensitivity analysis method to accommodate different missing mechanisms in cessation outcomes determined by self-report and urine validation results.

**Methods:**

We propose a two-stage imputation procedure, allowing survey and urine data to be missing under different mechanisms. The motivating data were from a tobacco cessation trial examining the effects of the extended vs. standard Quit and Win contests and counseling vs. no counseling under a 2-by-2 factorial design. The primary outcome was 6-month biochemically verified tobacco abstinence.

**Results:**

Our proposed method covers a wide spectrum of missing scenarios, including the widely adopted “missing = smoking” imputation by assuming a perfect smoking-missing correlation (an extreme case of MNAR), the MAR case by assuming a zero smoking-missing correlation, and many more in between. The analysis of the data example shows that the estimated effects of the studied interventions are sensitive to the different missing assumptions on the survey and urine data.

**Conclusions:**

Sensitivity analysis has played a crucial role in assessing the robustness of the findings in clinical trials with missing data. The proposed method provides an effective tool for analyzing missing data introduced at two different stages of outcome assessment, the self-report and validation time. Our methods are applicable to trials studying biochemically verified abstinence from alcohol and other substances.

**Electronic supplementary material:**

The online version of this article (10.1186/s12874-018-0635-2) contains supplementary material, which is available to authorized users.

## Background

Cigarette smoking is a risk factor for morbidity and mortality in the US and around the world [1–4]. Smoking cessation studies usually encourage cessation and provide either behavioral (e.g., counseling) or pharmaceutical (e.g., nicotine gum) interventions or both. In smoking cessation studies, missing binary abstinence outcomes (i.e., quit or not quit) are very common. These missing outcomes may lead to bias or weakened statistical power in estimating the effect of the studied intervention. Choosing appropriate statistical methods to handle binary missing data has been a continuing source of controversy [5].

The choice of methods to deal with missing data would depend on assumptions about the missing mechanism [6]. Data are referred to as being missing at random (MAR) if the missing status (yes or no) is not related to the missing value itself, but can be dependent on some other observed variables. Data are referred to as being missing not at random (MNAR) or nonignorable missing if the probability of missing depends on the missing value. It is well known that from the observed data alone, it is impossible to distinguish between MAR and MNAR. Therefore, statistical analyses based on one specific missing mechanism, such as the popular MAR assumption, could lead to misleading conclusions if it turns out that the missing is not at random. For example, consider a trial to incentivize smokers to quit smoking. Missing data due to a non-response in surveys or as a result of dropouts could be dependent on the smoking status of the participants, which renders the missing mechanism to be not at random. Sensitivity analysis can play a crucial role in assessing the robustness of the findings in clinical trials with missing data [7]. In this paper, we aim to study sensitivity analysis methods for analyzing smoking cessation outcome data under various missing data mechanisms including MAR and MNAR.

In the literature, the standard procedure used in smoking cessation trials is to assume that all non-respondents are smoking (referred to as the “missing = smoking” method hereinafter), which is a special case of single imputation under the MNAR assumption. Based on Jackson et al. [8], around 80% of reports of smoking cessation trials adopt this assumption. However, this simple imputation approach has been shown to lead to potentially biased results [8–12]. Other common single imputation methods frequently used in smoking cessation trials include the last observation carried forward (LOCF), the baseline observation carried forward (BOCF) and imputations based on predicted values from a regression model or the expectation-maximization (EM) algorithm [13]. In addition, Barnes and colleagues [14] used a multiple imputation procedure with the propensity score matching method to impute missing smoking status.

Hedeker and colleagues [12] demonstrated that the simple missing = smoking imputation is essentially based on the assumption that the missing status and the smoking status have a perfect correlation (*r* = 1) or, equivalently, the odds ratio (OR) between missing and smoking equals positive infinity (*OR* = +∞). They developed both simple and multiple imputation approaches based on more relaxed assumptions which allow different levels of correlation between smoking and missing status. Although their imputation method provides a more flexible and useful alternative to the simple missing = smoking method and has been applied in various trials [8, 13], this method cannot be directly applied to data with missing values generated from multiple sources or stages. For example, when cessation outcome is determined by self-report survey data followed by a urine validation test, both a non-respondent survey and a missing urine sample can lead to missing cessation outcomes. In this case, an imputation procedure designed to account for missing data generated from two different stages, the survey collection stage and the urine collection stage, would be preferable. A naive imputation approach for dealing with this type of missing data would treat only subjects who confirmed their abstinence by both self-report and a urine sample with a negative result as a treatment success (i.e., biochemically verified self-reported abstinence). All the other subjects including those who either failed to complete the survey or failed to provide the urine sample for confirmation of self-reported abstinence would be considered as treatment failures (i.e., not achieving abstinence). Note that this naive approach is an extreme case under the MNAR assumption, assuming a perfect correlation between survey missing and self-report failure and a perfect correlation between urine missing and urine-verified failure among people who self-reported abstinence. Hence, it does not have the flexibility to accommodate different levels of correlation between survey missing and self-report failure or between urine missing and urine-verified failure. In this paper, we extend Hedeker et al.’s method [12] to a two-stage imputation procedure to take into account missing data in either the self-report or the urine verification stages.

The rest of the article is organized as follows. In Section 2, we first introduce a randomized controlled trial of college smokers [15] which motivated this research, and then, we introduce a sensitivity analysis method using a two-stage imputation procedure for missing abstinence data at the self-reporting and subsequent biochemical verification stages. In Section 3, we report the sensitivity analysis result of the college smokers study. Some discussions and concluding remarks can be found in Sections 4 and 5, respectively.

## Methods

### Aim, design, and setting

The data motivating this research were collected from 1217 subjects enrolled in a smoking cessation randomized clinical trial entitled “Enhanced quit and win contests to improve smoking cessation among college students” (henceforth referred to as the “Enhanced Quit & Win” study) during the academic years 2010–2013. This study utilized a two-by-two factorial design to examine the marginal effect of two distinct interventions: the impact of multiple vs. single Quit & Win contests and the effect of the Motivational and Problem Solving counseling (MAPS) counseling vs. no counseling on smoking cessation among college smokers. Specifically, participants were randomly assigned to one of four groups: (1) single contest (denoted by Tx1, *n* = 306), (2) single contest plus counseling (Tx2, *n* = 296), (3) multiple contests (Tx3, *n* = 309), and (4) multiple contests plus counseling (Tx4, *n* = 306). The primary cessation outcome was measured at 6 months post-randomization when all participants were encouraged to complete an online survey to report their smoking status and other tobacco use in the past 30 days. Only people who reported no tobacco use in the past 30 days were invited to provide urine to biochemically (cotinine assay) confirm their self-reported abstinence. Both self-reported abstinence and biochemically verified abstinence were of interest. The study design and the characteristics of participants are described in greater detail in the parent study manuscript [15]. This trial was registered at ClinicalTrials.gov as number NCT01096108.

### Sensitivity analysis using two-stage imputation

As we described earlier, the missing data in the Enhanced Quit & Win study occurred at two different stages: the survey collection stage and the urine verification stage. A common and conservative imputation approach for dealing with such two-stage missing data would treat only subjects who self-reported abstinence and provided a urine sample which confirmed the abstinence as a treatment success (i.e., biochemically verified abstinence). All the other subjects including those who either failed to complete the survey or failed to provide urine would be considered as a treatment failure. This is analogous to the missing = smoking method for one-stage missing data. Note that this approach is an extreme case of the single imputation approach under the not missing at random (MNAR) assumption, assuming a perfect correlation between the survey missing and self-report failure, or equivalently an infinite odds ratio between the two (denoted by *OR*_1_ = ∞), and at the same time a perfect correlation between the urine missing and urine-verified failure (denoted by *OR*_2_ = ∞).

In this paper, we propose a two-stage imputation approach under the MNAR assumption, which takes into account the two-stage missing process and allows (1) different levels of correlation between the survey missing and self-report failure (i.e., varying *OR*_1_) and (2) different levels of correlation between the urine missing and urine-verified failure among those who self-reported abstinence (i.e., varying *OR*_2_). This can be considered as an extension of the imputation method in Hedeker et al. [12] for one-stage missing data to a two-stage missing data situation. In this section, we present a two-stage imputation approach conducted on a summary or aggregated data basis.

#### The one-stage imputation method by Hedeker et al. [12] for the self-report data

We first introduce some general notation. We code “tobacco use status”, the binary dependent variable as 1 = used tobacco/failure and 0 = did not use tobacco/abstinence and “missing status”, the binary indicator of whether the data is missing or not as 1 = missing and 0 = observed. Let *j* = 1, 2, 3, 4 index the four treatment groups, Tx1 to Tx4, respectively. Let subjects be indexed by *i* = 1, 2, …, *n*_*j*_, where *n*_*j*_ denotes the total number of subjects in treatment group Tx_*j*_. Since we propose to perform imputations within each treatment, in the sequel we omit *j* from all symbols to simplify notation. Moreover, we use superscripts 11, 12, 21, and 22 to denote the four entries of the two-by-two table between the tobacco use status and missing status, as illustrated in Table 1. Note that, in the second row of Table 1, only the total number of individuals with missing data, *n*^2.^, can be observed; the abstinence statuses of these people, *n*^21^ and *n*^22^ (the second row in Table 1) are unknown and need to be estimated. Furthermore, in the summation row of Table 1, the total number of abstinence (denoted by *n*^.1^) and the total number of failure (denoted by *n*^.2^) are also unknown. Note that the ‘dot’ in the superscripts indicates summation over a row or column.

**Table 1:** Two-by-two table of tobacco use status by missing for self-report data

	Self-report tobacco use status		
Missing status of self-report data	Abstinence	Failure	Total
Observed	*n* ^11^	*n* ^12^	*n* ^1.^
Missing	***n*** ^**21**^	***n*** ^**22**^	*n* ^2.^
Total	***n*** ^***.*1**^	***n*** ^***.*2**^	*n*

Bolded entries indicate values that are not observable

Following Hedeker et al. [12], in order to impute the numbers for abstinence and failure for participants with missing survey data (*n*^21^ and *n*^22^), we will assume an odds ratio for the missing survey status and self-report tobacco use status (*OR*_1_) to reflect the strength of correlation between them (denoted by *r*_1_). Note that the widely adopted missing = smoking method corresponds to the situation of *r*_1_ = 1 or *OR*_1_ = ∞. In that case, *n*^21^ is imputed with 0 and *n*^22^ is imputed with *n*^2.^. More generally, we have

1 $$ {OR}_1=\frac{\left({n}^{22}/{n}^{21}\right)}{\left({n}^{12}/{n}^{11}\right)},\mathrm{or}\ \mathrm{equivalently}\ \frac{n^{22}}{n^{21}}={OR}_1\frac{n^{12}}{n^{11}}, $$

and then it can be shown that the unobserved values, *n*^21^ and *n*^22^ can be imputed with the assumed *OR*_1_ by:

$$ {n}^{22}={n}^{2.}\frac{OR_1\ast Odds}{1+{OR}_1\ast Odds}=\pi {n}^{2.}\ \mathrm{and}\ {n}^{21}={n}^{2.}-{n}^{22}, $$

where *Odds* is the odds of tobacco using among survey respondents and can be calculated from the observed survey data by $\frac{n^{12}}{n^{11}}$; and $\pi =\frac{OR_1\ast Odds}{1+{OR}_1\ast Odds}$ is a multiplicative factor relating *n*^22^ to *n*^2.^.

Participants who do not respond or are lost to follow-up in a smoking cessation study may differ from those who are retained in the study with regard to their smoking status. We often expect that the odds of tobacco use among non-respondents is equal to or higher than that of respondents (i.e., *OR*_1_ ≥ 1), especially in studies where people are incentivized to quit as in the Enhanced Quit & Win study. Note that a larger *OR*_1_ would imply a stronger relationship between missing and tobacco use.

#### The two-stage imputation method for the urine-verified data

**Fig. 1:**
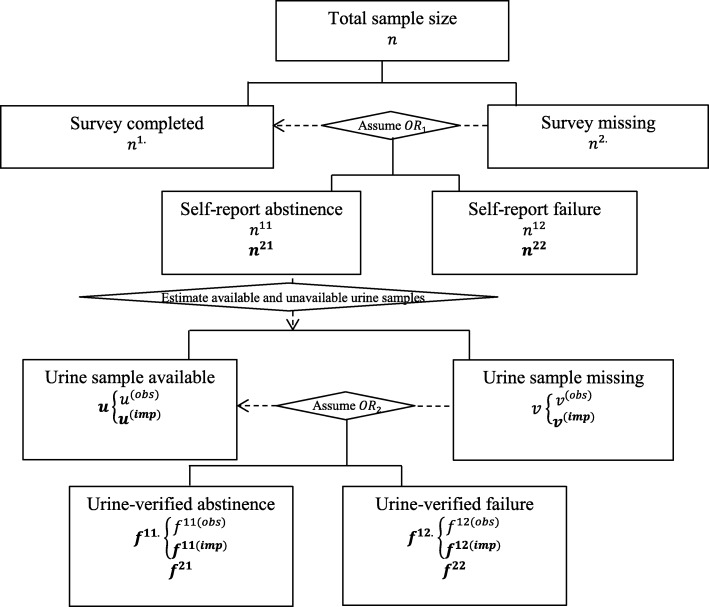
Data structure and notation for a single treatment group. Note *n* is the total sample size, *n*^1.^ is the number of survey respondents, and *n*^2.^ is the number of survey non-respondents. Then among survey respondents, denote *n*^11^ as the number of observed self-report abstinence and *n*^21^ as the number of imputed self-report abstinence. Similarly, *n*^12^ and *n*^22^ represent the number of observed failures and imputed failures based on the self-report data, respectively. For the urine samples, *u*^(*obs*)^ and *u*^(*imp*)^ represent the number of observed and estimated (based on the imputed survey data) urine samples being provided; similar notations, *v*^(*obs*)^ and *v*^(*imp*)^ are used for the number of unavailable urine samples. For the urine data, analogous notations are defined as for the survey data except for using *f*, instead of *n*, to denote the numbers of subjects under different conditions (with the superscript 11, 12, 21, and 22 having the same meaning). In addition, we used *f*^11(*imp*)^ to denote the abstinence and *f*^12(*imp*)^ to denote the failure obtained from the estimated available urine samples *u*^(*imp*)^).Then we combined the *f*^11(*obs*)^ and *f*^11(*imp*)^ to obtain the number of urine-verified abstinence *f*^11.^ among the urine samples what were actually provided or could have been provided if all surveys were completed, whereas combined *f*^12(*obs*)^ and *f*^12(*imp*)^ to obtain the urine-verified failure *f*^12.^.Denote *OR*_1_ as the assumed odds ratio between missing and smoking for self-report data and *OR*_2_ for urine data. Dashed lines indicate where missing data are reallocated based on certain assumptions or estimations. Bolded notation denotes values that are not observed

When estimating biochemically verified abstinence, more complex conditions should be considered since missing data can be present at both the survey and the urine verification stage. Without specification, the notation for the survey data are the same as those defined previously (see Table 1 and the top half of Fig. 1). Some additional notations, shown in the lower half of Fig. 1, specific to the urine data are defined as follows. Let *u*^(*obs*)^ and *u*^(*imp*)^ denote the number of urine samples provided by people who self-reported abstinence (*n*^11^) and the estimated number of urine samples that could be collected from people who would report abstinence if they did not fail to respond to the survey (*n*^21^), respectively; similarly, *v*^(*obs*)^ and *v*^(*imp*)^ are used for the number of missing urine samples of *n*^11^ and *n*^21^, respectively. For the urine-verified abstinence outcome, similar notation is defined as for the self-report abstinence outcome except for using *f*, instead of *n*. The superscript 11, 12, 21, and 22 have the same meaning as those for *n*. In addition, we use *f*^11(*obs*)^ to denote the number of urine-verified abstinence and *f*^12(*obs*)^ the number of urine-verified failures obtained from people who actually provided urine samples, and we have *u*^(*obs*)^ = *f*^11(*obs*)^ + *f*^12(*obs*)^.Similarly, we use *f*^11(*imp*)^ to denote the number of urine-verified abstinence and *f*^12(*imp*)^ the number of urine-verified failures obtained from the estimated available urine samples, and we have *u*^(*imp*)^ = *f*^11(*imp*)^ + *f*^12(*imp*)^. Then we combine *f*^11(*obs*)^and *f*^11(*imp*)^ to obtain the total number of participants with urine-verified abstinence *f*^11.^, among the urine samples what were actually provided or could have been provided if there were no surveys missing; similarly, we combine *f*^12(*obs*)^ and *f*^12(*imp*)^ to obtain the total number of urine verified failures *f*^12.^.

Based on the previous imputation results for missing data at the survey stage, self-report abstinence (*n*^11^*, n*^21^) and failed abstinence (*n*^12^*, n*^22^) have been generated based on the imputed survey data under the assumed *OR*_1_ within each treatment. Next, we proceed to estimate urine-verified abstinence or failure under the assumed *OR*_2_ for the imputed, “complete” self-report data. Prior to imputing missing urine sample data, the numbers of subjects who would have provided urine samples (*u*^(*imp*)^) or would not provide urine samples (*v*^(*imp*)^) among survey non-respondents need to be estimated. One can assume that the urine missing rate among survey non-respondents, compared with respondents, varies by a known factor λ (λ > 0), that is

2 $$ \frac{u^{(imp)}}{v^{(imp)}}=\lambda \frac{u^{(obs)}}{v^{(obs)}} $$

Consequently, the number of available (*u*^(*imp*)^) and unavailable (*v*^(*imp*)^) urine samples among imputed self-report abstinence cases can be calculated based on Equation (2) and the fact that *u*^(*imp*)^ + *v*^(*imp*)^ = *n*^21^.

Similarly, one can assume that, compared to the actually provided urine samples, the urine-verified abstinence rate among the urine samples that could have been provided if the survey were completed, varies by a known factor *η* (*η* > 0):

3 $$ \frac{f^{11(imp)}}{f^{12(imp)}}=\eta \frac{f^{11(obs)}}{f^{12(obs)}}. $$

Therefore, the number of urine-verified abstinence (*f*^11(*imp*)^) and failure (*f*^12(*imp*)^) among imputed self-report abstinence cases people can be estimated based on Equation (3) and the fact that *f*^11(*imp*)^ + *f*^12(*imp*)^ = *u*^(*imp*)^.

We then can calculate the total number of urine-verified abstinence cases by *f*^11.^ = *f*^11(*obs*)^ + *f*^11(*imp*)^ and the urine-verified failure by *f*^12.^ = *f*^12(*obs*)^ + *f*^12(*imp*)^ among all the “available” urine samples (including actually observed or imputed). Up to this point, the urine-verified abstinence (*f*^21^) and urine-verified failure (*f*^22^), among people whose urine was not actually provided (*v*^(*obs*)^) or would not be provided even if their survey data were completed (*v*^(*imp*)^), have not yet been imputed. Next, we use the fact that *v* = *f*^22^ + *f*^21^ = *v*^(*obs*)^ + *v*^(*imp*)^ and propose a similar imputation procedure for the urine missing data as for the survey missing data described in the previous subsection as follows:

$$ \frac{f^{22}}{f^{21}}={OR}_2\frac{f^{12.}}{f^{11.}}\ \mathrm{or}\ \mathrm{equivalently},{f}^{22}=v\frac{OR_2\ast {Odds}^{\prime }}{1+\left({OR}_2\ast {Odds}^{\prime}\right)}=v{\pi}^{\prime }, $$

where the second equality follows from Equation (3), and *Odds*^′^ and *π*^′^ are the odds of tobacco use and probability of tobacco use among people who provided urine sample, respectively. The overall number of participants with urine-verified abstinence can then be obtained by simply adding *f*^11.^ and *f*^21^, and similarly, the overall number of urine-verified failure is *f*^12.^ + *f*^22^. After all the above steps are completed for each treatment arm, we can estimate the various treatment effects based on the imputed data.

For the Enhanced Quit & Win data, we assumed a series of ≥1 values for *OR*_1_ and *OR*_2_ (1, 2, 3, 4, 5, and positive infinity) and that *λ* = *η* = 1 for the ease of presentation, but certainly more values can be examined for these parameters in the sensitivity analysis. SAS Version 9.4 (SAS Institute Inc., Cary, NC, USA) was used for all analyses and the SAS computing code for the proposed two-stage imputation method is provided in the Additional file 1: Supplementary Material.

## Results

### Summary of missing data

**Fig. 2:**
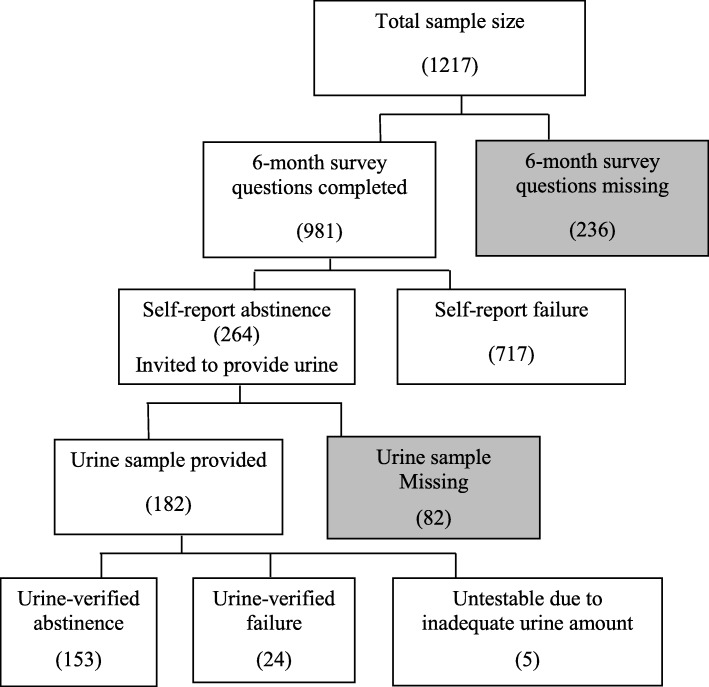
Missing data in 6-month abstinence outcomes of the Enhanced Quit & Win study (subjects with missing abstinence data are shaded)

Figure 2 shows the summary of the 6-month abstinence outcomes and missing data. Of the 1217 randomized participants, 981 (81%) completed the 6-month survey and 236 (19%) did not. Among the 981 survey completers, 264 (27%) self-reported tobacco abstinence. Among the 264 participants who self-reported abstinence, 182 (69%) provided urine. Among the 182 participants who provided urine samples, 5 were not of adequate amount for testing and 153 (84%) were biochemically confirmed as abstinent.

Table 2 presents the differential missing data patterns across treatment arms and intervention conditions by both survey missing and urine missing. Note that the five missing urine test results due to inadequate urine amount were assumed to have the same distribution as the other 177 urine samples (86% verified abstinence and 14% verified failure) and added to the corresponding columns in Table 2. We found that the no counseling groups, Tx1 and Tx3, had significantly (*p* = 0.003) lower survey missing rates (15.4 and 16.5%, respectively and 15.9% for the combined group) than the two counseling arms, Tx2 and Tx4 (22.6 and 23.2%, respectively and 22.9% for the combined group), whereas the single- and multiple-contests groups were found to have similar survey missing rates (*p* = 0.798). The urine missing rate was similar between the single- and multiple-contests groups and between the counseling and no counseling groups (both *p*s > 0.05).

**Table 2:** Summary of 6-month self-reported and urine verified abstinence and missing data by treatment arms and by type of intervention

		Self-report abstinence and survey missing at 6 months			Urine-verified abstinence and urine missing at 6 months		
Treatment group	Total *N*	Self-report abstinence *n* (%)	Self-report failure *n* (%)	Survey question missing *n* (%)	Urine-verified abstinence *n* (%^a^)	Urine-verified failure *n* (%^a^)	Urine missing *n* (%^a^)
By treatment arm							
Tx1	306	65 (21.2%)	194 (63.4%)	47 (15.4%)	38 (58.5%)	6 (9.2%)	21 (32.3%)
Tx2	296	59 (19.9%)	170 (57.4%)	67 (22.6%)	34 (57.6%)	6 (10.2%)	19 (32.2%)
Tx3	309	61 (19.7%)	197 (63.8%)	51 (16.5%)	35 (57.4%)	6 (9.8%)	20 (32.8%)
Tx4	306	79 (25.8%)	156 (51.0%)	71 (23.2%)	50 (63.3%)	7 (8.9%)	22 (27.8%)
By intervention							
No counseling (Tx1 + Tx3)	615	126 (20.5%)	391 (63.6%)	98 (15.9%)	73 (57.9%)	12 (9.5%)	41 (32.5%)
Counseling (Tx2 + Tx4)	602	138 (22.9%)	326 (54.2%)	138 (22.9%)	84 (60.9%)	13 (9.4%)	41 (29.7%)
Single contest (Tx1 + Tx2)	602	124 (20.6%)	364 (60.5%)	114 (18.9%)	72 (58.1%)	12 (9.7%)	40 (32.2%)
Multiple contests (Tx3 + Tx4)	615	140 (22.8%)	353 (57.4%)	122 (19.8%)	85 (60.7%)	13 (9.3%)	42 (30.0%)
Overall	1217	264 (21.7%)	717 (58.9%)	236 (19.4%)	157 (59.5%)^b^	25 (9.5%)^b^	82 (31.1%)

Tx1: single contest + no counseling; Tx2: single contest + counseling; Tx3: multiple contests + no counseling; Tx4: multiple contest + counseling

^a^Percentage out of those who self-reported abstinence

^b^Five subjects whose urine samples were not of adequate amount for testing. These 5 missing urine test results were assumed to have the same distribution as the rest 177 urine samples (86% verified abstinence and 14% verified failure) and added to the two columns accordingly

### Self-report abstinence outcome

The imputation results of the self-report abstinence outcome are summarized in Table 3. As a comparison, we also present the results from a complete case only analysis, where only subjects with no missing survey or urine were included. We can prove that the abstinence rate decreases as *OR*_1_ increases. As expected, the estimated abstinence rates and treatment effect based on the imputed data under the MAR assumption (i.e., *OR*_1_ = 1) are the same as those based on the complete case only analysis. However, the statistical significance is stronger (smaller *p*) in the former as more data are utilized. Under the MAR assumption, the estimated treatment effect of counseling vs. no counseling is significant (OR for abstinence = 1.31, *p* = 0.034); however, as *OR*_1_increases, the estimated treatment effect becomes less significant (all *p*s > 0.05 for *OR*_1_ ≥ 2), indicating that this treatment effect is sensitive to different assumed values of *OR*_1_. On the contrary, the estimated treatment effects of multiple vs. single contest are all close to 1.16 (all *p*s > 0.05), indicating that this treatment effect estimation was robust to different assumed values of *OR*_1_. This phenomenon can be explained by the different survey missing rate between the counseling and no counseling groups, but not between the multiple and single contest groups (see the left panel in Table 2).

**Table 3:** Summary of imputation results for self-report abstinence assuming different levels of association between the survey missing status and self-report abstinence

	Counseling vs. no counseling				Multiple vs. single contests			
*OR*_1_	Counseling Tx2 + Tx4	No counseling Tx1 + Tx3	Estimated treatment effect (odds ratio for abstinence)	*P-*value	Multiple contests Tx3 + Tx4	Single contest Tx1 + Tx2	Estimated treatment effect (odds ratio for abstinence)	*P*-value
Complete case only	29.7%	24.4%	1.31	.058	28.4%	25.4%	1.16	.291
1	29.8%	24.4%	1.31	.034	28.6%	25.4%	1.18	.212
2	27.0%	22.7%	1.26	.086	26.2%	23.4%	1.16	.251
3	25.8%	22.0%	1.23	.125	25.2%	22.5%	1.16	.275
4	25.1%	21.7%	1.21	.154	24.7%	22.1%	1.15	.290
5	24.7%	21.5%	1.20	.175	24.3%	21.8%	1.15	.301
⁞								
+∞	22.9%	20.5%	1.15	.303	22.8%	20.6%	1.14	.359

*OR*_1_: odds ratio between missing and tobacco use status for self-report data, where *OR*_1_ = 1 corresponds to the situation when missing is independent of tobacco use and *OR*_1_ = positive infinity (+∞) corresponds to the situation when missing = smoking; Tx1: single contest + no counseling; Tx2: single contest + counseling; Tx3: multiple contests + no counseling; Tx4: multiple contest + counseling. *P*-values are based on the Chi-square test

### Urine-verified abstinence outcome

The results obtained from the imputed urine-verified abstinence data were summarized in Table 4. By considering all the combinations of *OR*_1_ and *OR*_2_, each ranging from 1 to 5 and positive infinity, we found that the abstinence rate decreases as the assumed level of dependence between missing and tobacco use, *OR*_1_ or *OR*_2_ increases, as expected. Notice that the abstinence rates for the two studied conditions were found consistently higher than their corresponding control groups in all scenarios (i.e., the estimated treatment effect as indicated as odds ratios of abstinence are all > 1).

**Table 4:** Summary of imputation results for urine-verified abstinence assuming different levels of association between missing and abstinence

		Counseling vs. no counseling				Multiple vs. single contests			
*OR*_1_	*OR*_2_	Counseling Tx2 + Tx4	No counseling Tx1 + Tx3	Estimated treatment effect (odds ratio for abstinence)	*P*-value	Multiple contests Tx3 + Tx4	Single contest Tx1 + Tx2	Estimated treatment effect (odds ratio for abstinence)	*P*-value
1	1	25.8%	20.9%	1.31	.046	24.8%	21.8%	1.18	.212
	2	24.8%	20.1%	1.32	.046	23.9%	20.9%	1.19	.204
	3	24.1%	19.4%	1.32	.047	23.3%	20.2%	1.20	.200
	4	23.6%	18.9%	1.32	.047	22.7%	19.7%	1.20	.197
	5	23.1%	18.5%	1.32	.047	22.2%	19.2%	1.20	.195
	+∞	18.1%	14.1%	1.35	.058	17.4%	14.8%	1.22	.209
2	1	23.3%	19.5%	1.26	.102	22.7%	20.0%	1.18	.249
	2	22.5%	18.7%	1.26	.101	21.9%	19.2%	1.18	.241
	3	21.9%	18.1%	1.27	.100	21.3%	18.6%	1.19	.236
	4	21.3%	17.6%	1.27	.100	20.8%	18.1%	1.19	.233
	5	20.9%	17.2%	1.27	.100	20.4%	17.7%	1.19	.231
	+∞	16.4%	13.1%	1.30	.109	15.9%	13.6%	1.21	.244
3	1	22.3%	18.9%	1.23	.143	21.9%	19.3%	1.17	.272
	2	21.5%	18.2%	1.24	.140	21.1%	18.5%	1.17	.264
	3	20.9%	17.6%	1.24	.139	20.5%	17.9%	1.18	.258
	4	20.4%	17.1%	1.24	.137	20.0%	17.4%	1.18	.255
	5	20.0%	16.7%	1.25	.137	19.6%	17.0%	1.19	.253
	+∞	15.7%	12.8%	1.27	.143	15.3%	13.1%	1.20	.264
4	1	21.8%	18.6%	1.22	.171	21.4%	18.9%	1.16	.287
	2	21.0%	17.9%	1.22	.168	20.6%	18.2%	1.17	.278
	3	20.4%	17.3%	1.23	.165	20.0%	17.6%	1.17	.272
	4	19.9%	16.8%	1.23	.164	19.6%	17.1%	1.18	.269
	5	19.5%	16.4%	1.23	.163	19.2%	16.7%	1.18	.266
	+∞	15.3%	12.6%	1.26	.166	15.0%	12.8%	1.20	.277
5	1	21.4%	18.4%	1.21	.192	21.1%	18.7%	1.16	.297
	2	20.7%	17.7%	1.21	.188	20.3%	17.9%	1.17	.288
	3	20.1%	17.1%	1.22	.185	19.8%	17.4%	1.17	.282
	4	19.6%	16.6%	1.22	.183	19.3%	16.9%	1.18	.279
	5	19.2%	16.3%	1.22	.181	18.9%	16.5%	1.18	.276
	+∞	15.1%	12.4%	1.25	.183	14.8%	12.7%	1.20	.285
+∞	1	19.8%	17.6%	1.16	.315	19.7%	17.7%	1.15	.352
	2	19.1%	16.9%	1.17	.306	19.0%	16.9%	1.15	.342
	3	18.6%	16.3%	1.17	.300	18.5%	16.4%	1.16	.335
	4	18.1%	15.9%	1.17	.295	18.0%	15.9%	1.16	.331
	5	17.8%	15.5%	1.18	.292	17.7%	15.6%	1.16	.328
	+∞	14.0%	11.9%	1.20	.278	13.8%	12.0%	1.18	.333

*OR*_1_: odds ratio between missing and tobacco use status for self-report data; *OR*_2_: odds ratio between urine missing and urine-verified failure among those who self-reported abstinence; Tx1: single contest + no counseling; Tx2: single contest + counseling; Tx3: multiple contests + no counseling; Tx4: multiple contests + counseling. *P*-values are based on the Chi-square test

As shown in the upper-left corner of Table 4, significant treatment effects were estimated for the counseling group when both *OR*_1_ and *OR*_2_ were small. Otherwise, there seemed to be no significant treatment effects for the counseling or the multiple contests groups under different combinations of *OR*_1_ and *OR*_2_. We also found that the estimated treatment effect of counseling vs. no counseling is more sensitive to the assumed level of dependence between the survey missing and self-report abstinence, but less sensitive to the assumed level of dependence between the urine missing and urine-verified abstinence. For the estimated treatment effect of the multiple- vs. single-contest, we observed no obvious pattern, no matter what values were assumed for *OR*_1_ or *OR*_2_. This can be explained by the comparable survey and urine missing rates between the two contest groups as shown in Table 2. We performed additional sensitivity analysis by assuming that survey non-respondents would be less likely to provide urine than survey respondents (λ = 0.5). Results (shown in Additional file 1: Table S1) are consistent with the results reported above which are based on the equal urine missing rate assumption (λ = 1).

## Discussion

In many smoking cessation studies, researchers are interested in biochemically verified abstinence (e.g., urine cotinine verified abstinence). To conserve resources, it is common to only invite people who self-report abstinence to provide biochemical samples to validate self-reported abstinence. Hence, missing data can be present at either the survey completion stage or the biochemical sampling stage. The imputation approaches presented in this paper take into account this two-stage missing data challenge and describes a two-step imputation approach allowing the survey missing and biochemical sample missing to have different missing mechanisms. Our proposed imputation approach includes both the missing = smoking imputation (an extreme case of MNAR) and the MAR imputation as special cases, hence providing a more thorough sensitivity analysis result than any simple imputation method alone. The estimated effect of the treatments tested in the Enhanced Quit & Win study were sensitive to the different missing mechanisms depending on the differential missing data patterns across treatment arms. Although the overall results were not universally impacted, these findings demonstrate that the use of one simple imputation method alone could result in misleading conclusions regarding a treatment effect estimate.

There has been a debate regarding whether treatment should be adjusted or stratified in the imputation models. Jackson et al. [16] adjusted for treatment in their imputation model since treatment was found to be associated with the missing status and predicted missing outcomes. Alternatively, in this paper, we performed imputations stratified by treatment rather than adjusting for treatment in the model [17, 18]. Although some researchers may argue that this may overestimate the treatment effect [16], it has not been demonstrated by the preponderance of evidence. Research with more data examples to investigate the difference between these two strategies is certainly warranted.

In this paper, all the imputations were performed on aggregated data. In other words, no individual-level variation has been considered. Currently, we are working on extending the proposed imputation approach for aggregated data to take into account the uncertainty in the individual probability of tobacco use as in multiple imputations. One advantage of the imputations based on aggregated data is the ease of computing, while the multiple imputations approach is expected to give more conservative results as individual level variability is taken into account in the estimation of treatment effect. Also in this paper, we focus on the analysis of cessation outcome at a single time point. However, with repeatedly measured outcomes, longitudinal data analysis methods for dealing with missing data could be considered. [12, 19–21]. Note that our proposed methods are applicable to various tobacco or other substance use trials where the treatment goal is biochemically verified self-reported abstinence.

## Conclusions

The proposed two-stage imputation method provides an effective sensitivity analysis tool for analyzing missing data introduced at two different stages of outcome assessment, the self-report and validation time, frequently encountered in tobacco cessation studies. Our methods are also applicable to trials studying biochemically verified abstinence from other substance use such as alcohol and recreational drugs.

## Additional file

Additional file 1: SAS Computing Code for Analyzing Enhanced Quit & Win Data. **Table S1.** Summary of imputation results for urine-verified abstinence assuming different levels of association between missing and abstinence when λ = 0.5. (DOCX 58 kb)

## Abbreviations

MAR: Missing at random
MCAR: Missing completely at random
MNAR: Missing not at random
LOCF: Last observation carried forward
BOCF: Baseline observation carried forward
EM: Expectation-maximization
OR: Odds ratio
MAPS: Motivational and problem solving counseling
