# Supplementary material for: Analysis of self-report and biochemically verified tobacco abstinence outcomes with missing data: a sensitivity analysis using two-stage imputation
Source: BMC Med Res Methodol. 2018 Dec 18;18:170. doi: 10.1186/s12874-018-0635-2 (PMC6299502; doi:10.1186/s12874-018-0635-2)
Supplement: Supplementary file 1 — SAS Computing Code for Analyzing Enhanced Quit & Win Data. Table S1. Summary of imputation results for urine-verified abstinence assuming different levels of association between missing and abstinence when λ = 0.5. (DOCX 58 kb) [file 12874_2018_635_MOESM1_ESM.docx]

Analysis of Self-Report and Biochemically Verified Tobacco Abstinence Outcomes with Missing Data:

A Sensitivity Analysis Using Two-Stage Imputation

Yiwen Zhang, ^*^Xianghua Luo, Chap T. Le, Jasjit S. Ahluwalia, Janet L. Thomas

**Additional file 1: Supplementary material**

**SAS Computing Code for Analyzing Enhanced Quit & Win Data**

*******************************************

*** Set macro variables for later usage ***

*******************************************;

/*Total sample size for T1, T2, T3 and T4 group*/

%let n_1=306;

%let n_2=296;

%let n_3=309;

%let n_4=306;

/*Missing number for T1, T2, T3 and T4 group*/

%let n2_1=47;

%let n2_2=67;

%let n2_3=51;

%let n2_4=71;

/*Sample size for counselling, non-counselling, multiple-contests

and single-contest groups*/

%let couns=%eval(&n_2+&n_4);

%let nocouns=%eval(&n_1+&n_3);

%let mcont=%eval(&n_3+&n_4);

%let scont=%eval(&n_1+&n_2);

***********************************************************************

*** Code for self-reported data imputation and analysis starts here ***

***********************************************************************;

**data** temp1;

input n11 n12 n2;

datalines;

65 194 47

59 170 67

61 197 51

79 156 71

;

**run**;

**data** temp1;

set temp1;

array pct_s(**5**) pct_s1-pct_s5;

array imput(**5**) imput1-imput5; /*n.1 in paper*/

odds=n12/n11; /*observed odds of failure*/

do i=**1** to **5**;

/*percentage (pct) of estimated success in missing data with OR1=1 to 5*/

pct_s(i)=**1**-i*odds/(**1**+i*odds);

/*total number of self-reported abstinence after imputation*/

imput(i)=pct_s(i)*n2+n11;

end;

cp_case=n11; /*self-reported abstinence for complete-case analysis*/

inf=n11; /*self-reported abstinence with OR1=infinity (inf)*/

keep imput1-imput5 inf cp_case;

**run**;

**proc** **transpose** data=temp1 out=temp2 prefix=T;**run**;

**data** temp3;

set temp2;

couns_pct=(T2+T4)/&couns; /*pct of abstinence in counselling group*/

ncouns_pct=(T1+T3)/&nocouns; /*pct of abstinence in no counselling group*/

mcont_pct=(T3+T4)/&mcont; /*pct of abstinence in multiple contest group*/

scont_pct=(T1+T2)/&scont; /*pct of abstinence in single contest group*/

/*odds ratio of counselling group*/

or_couns=((T2+T4)/(&couns-T2-T4))/((T1+T3)/(&nocouns-T1-T3));

/*odds ratio of contests group*/

or_cont=((T3+T4)/(&mcont-T3-T4))/((T1+T2)/(&scont-T1-T2));

if _NAME_="cp_case" then OR1=**0**; /*complete case analysis*/

if _NAME_="imput1" then OR1=**1**;

if _NAME_="imput2" then OR1=**2**;

if _NAME_="imput3" then OR1=**3**;

if _NAME_="imput4" then OR1=**4**;

if _NAME_="imput5" then OR1=**5**;

if _NAME_="inf" then OR1=**999**; /*OR1=infinity*/

/*complete case analysis*/

if OR1=**0** then do;

couns_pct=(T2+T4)/(&couns-&n2_2-&n2_4);

ncouns_pct=(T1+T3)/(&nocouns-&n2_1-&n2_3);

mcont_pct=(T3+T4)/(&mcont-&n2_3-&n2_4);

scont_pct=(T1+T2)/(&scont-&n2_1-&n2_2);

or_couns=((T2+T4)/((&couns-&n2_2-&n2_4)-T2-T4))/((T1+T3)/((&nocouns-&n2_1-&n2_3)-T1-T3));

or_cont=((T3+T4)/((&mcont-&n2_3-&n2_4)-T3-T4))/((T1+T2)/((&scont-&n2_1-&n2_2)-T1-T2));

end;

**run**;

**data** chisq_data;

input Group $ response $;

datalines;

Group1 yes

Group2 yes

Group1 no

Group2 no

;

**run**;

**%macro** chisq_pvalue(datain, /*input dataset*/

dataout, /*output dataset*/

gr1, /*nubmer of abstinence for the first group*/

gr2, /*number of abstinence for the second group*/

gr3, /*number of abstinence for the third group*/

gr4, /*number of abstinence for the fourth group*/

tot1, /*total number of the first and second group*/

tot2, /*total number of the third and fourth group*/

n_OR, /*assumed number of OR1 or OR2*/

OR); /*OR1 or OR2*/

data &dataout;

set &datain;

group1_y=&gr1.+&gr2.;

group2_y=&gr3.+&gr4.;

group1_n=&tot1.-group1_y;

group2_n=&tot2.-group2_y;

keep group1_y group2_y group1_n group2_n &OR.;

run;

proc transpose data=&dataout out=&dataout;

where &OR.=&n_OR.;

run;

data &dataout;

set &dataout;

if _NAME_="OR1" then delete;

if _NAME_="OR2" then delete;

run;

data &dataout;

merge &dataout chisq_data;

run;

proc sort data=&dataout;

by group response;

run;

proc freq data=&dataout noprint;

tables group*response/chisq;

weight col1;

output out=&dataout pchi;

run;

**%mend**;

/*counselling group*/

%***chisq_pvalue***(datain=temp3,dataout=p_0,gr1=T2,gr2=T4,gr3=T1,gr4=T3,tot1=%str(&couns-&n2_2-&n2_4),tot2=%str(&nocouns-&n2_1-&n2_3),n_OR=**0**,OR=or1);

%***chisq_pvalue***(datain=temp3,dataout=p_1,gr1=T2,gr2=T4,gr3=T1,gr4=T3,tot1=%str(&couns),tot2=%str(&nocouns),n_OR=**1**,OR=or1);

%***chisq_pvalue***(datain=temp3,dataout=P_2,gr1=T2,gr2=T4,gr3=T1,gr4=T3,tot1=%str(&couns),tot2=%str(&nocouns),n_OR=**2**,OR=or1);

%***chisq_pvalue***(datain=temp3,dataout=P_3,gr1=T2,gr2=T4,gr3=T1,gr4=T3,tot1=%str(&couns),tot2=%str(&nocouns),n_OR=**3**,OR=or1);

%***chisq_pvalue***(datain=temp3,dataout=P_4,gr1=T2,gr2=T4,gr3=T1,gr4=T3,tot1=%str(&couns),tot2=%str(&nocouns),n_OR=**4**,OR=or1);

%***chisq_pvalue***(datain=temp3,dataout=P_5,gr1=T2,gr2=T4,gr3=T1,gr4=T3,tot1=%str(&couns),tot2=%str(&nocouns),n_OR=**5**,OR=or1);

%***chisq_pvalue***(datain=temp3,dataout=p_inf,gr1=T2,gr2=T4,gr3=T1,gr4=T3,tot1=%str(&couns),tot2=%str(&nocouns),n_OR=**999**,OR=or1);

**data** couns_pvalue;

set p_0(in=a) p_1(in=b) p_2(in=c) p_3(in=d) p_4(in=e) p_5(in=f) p_inf(in=g);

if a then OR1=**0**;

if b then OR1=**1**;

if c then OR1=**2**;

if d then OR1=**3**;

if e then OR1=**4**;

if f then OR1=**5**;

if g then OR1=**999**;

**run**;

/*contest group*/

%***chisq_pvalue***(datain=temp3,dataout=p_0,gr1=T3,gr2=T4,gr3=T1,gr4=T2,tot1=%str(&mcont-&n2_3-&n2_4),tot2=%str(&scont-&n2_1-&n2_2),n_OR=**0**,OR=or1);

%***chisq_pvalue***(datain=temp3,dataout=p_1,gr1=T3,gr2=T4,gr3=T1,gr4=T2,tot1=%str(&mcont),tot2=%str(&scont),n_OR=**1**,OR=or1);

%***chisq_pvalue***(datain=temp3,dataout=P_2,gr1=T3,gr2=T4,gr3=T1,gr4=T2,tot1=%str(&mcont),tot2=%str(&scont),n_OR=**2**,OR=or1);

%***chisq_pvalue***(datain=temp3,dataout=P_3,gr1=T3,gr2=T4,gr3=T1,gr4=T2,tot1=%str(&mcont),tot2=%str(&scont),n_OR=**3**,OR=or1);

%***chisq_pvalue***(datain=temp3,dataout=P_4,gr1=T3,gr2=T4,gr3=T1,gr4=T2,tot1=%str(&mcont),tot2=%str(&scont),n_OR=**4**,OR=or1);

%***chisq_pvalue***(datain=temp3,dataout=P_5,gr1=T3,gr2=T4,gr3=T1,gr4=T2,tot1=%str(&mcont),tot2=%str(&scont),n_OR=**5**,OR=or1);

%***chisq_pvalue***(datain=temp3,dataout=p_inf,gr1=T3,gr2=T4,gr3=T1,gr4=T2,tot1=%str(&mcont),tot2=%str(&scont),n_OR=**999**,OR=or1);

**data** cont_pvalue;

set p_0(in=a) p_1(in=b) p_2(in=c) p_3(in=d) p_4(in=e) p_5(in=f) p_inf(in=g);

if a then OR1=**0**;

if b then OR1=**1**;

if c then OR1=**2**;

if d then OR1=**3**;

if e then OR1=**4**;

if f then OR1=**5**;

if g then OR1=**999**;

**run**;

****************************************************************

*** Imputation and analysis for self-reported data ends here ***

****************************************************************;

***************************************************************

*** Code for urine data imputation and analysis starts here ***

***************************************************************;

/*Let lambda=1 or 0.5 for sensitive analysis below*/

%let lambda=1;

%let lambda=0.5;

**data** utemp1;

set temp1;

/*imputed self-reported abstinence with OR1=1 to 5 and OR1=inf*/

n21_1=imput1-cp_case;

n21_2=imput2-cp_case;

n21_3=imput3-cp_case;

n21_4=imput4-cp_case;

n21_5=imput5-cp_case;

n21_inf=**0**;

input u_obs f11_obs f12_obs;

datalines;

44 38 6

40 34 6

41 35 6

57 50 7

;

**run**;

**data** utemp2;

set utemp1;

/*"provided" urine sample: observed + imputed self-report data*/

u_1=u_obs+n21_1*&lambda.*(u_obs/cp_case);

u_2=u_obs+n21_2*&lambda.*(u_obs/cp_case);

u_3=u_obs+n21_3*&lambda.*(u_obs/cp_case);

u_4=u_obs+n21_4*&lambda.*(u_obs/cp_case);

u_5=u_obs+n21_5*&lambda.*(u_obs/cp_case);

u_inf=u_obs+n21_inf*&lambda.*(u_obs/cp_case);

/*missing urine sample among self-reported abstinence*/

nu_1=imput1-u_1;

nu_2=imput2-u_2;

nu_3=imput3-u_3;

nu_4=imput4-u_4;

nu_5=imput5-u_5;

nu_inf=inf-u_inf;

/*"observed" urine abstience: observed + imputed self-report data*/

f11_1=f11_obs+n21_1*&lambda.*(u_obs/cp_case)*(f11_obs/u_obs);

f11_2=f11_obs+n21_2*&lambda.*(u_obs/cp_case)*(f11_obs/u_obs);

f11_3=f11_obs+n21_3*&lambda.*(u_obs/cp_case)*(f11_obs/u_obs);

f11_4=f11_obs+n21_4*&lambda.*(u_obs/cp_case)*(f11_obs/u_obs);

f11_5=f11_obs+n21_5*&lambda.*(u_obs/cp_case)*(f11_obs/u_obs);

f11_inf=f11_obs+n21_inf*&lambda.*(u_obs/cp_case)*(f11_obs/u_obs);

/*"observed" urine failure: observed + imputed self-report data*/

f12_1=u_1-f11_1;

f12_2=u_2-f11_2;

f12_3=u_3-f11_3;

f12_4=u_4-f11_4;

f12_5=u_5-f11_5;

f12_inf=u_inf-f11_inf;

**run**;

**%macro** urine_imput(datain, /*input dataset*/

dataout, /*output dataset*/

imput, /*imputed+observed self-reported abstinence*/

f12_, /*nubmer of "observed" urine failure*/

f11_, /*nubmer of "observed" urine abstinence*/

nu); /*nubmer of urine missingness*/

data &dataout.;

set &datain.;

array upct_s(**5**) upct_s1-upct_s5;

array uimput(**5**) uimput1-uimput5; /*f11.+f21 in paper*/

do i=**1** to **5**;

/*pct of estimated success among urine missing data*/

upct_s[i]=**1**-(i*&f12_./&f11_.)/(**1**+(i*&f12_./&f11_.));

/*total number of urine abstinence for each group after imputation*/

uimput(i)=upct_s[i]*&nu.+&f11_.;

end;

inf=&imput-(&nu+&f12_);

keep uimput1-uimput5 inf;

run;

proc transpose data=&dataout. out=&dataout. prefix=T;run;

data &dataout.;

set &dataout.;

couns_pct=(T2+T4)/&couns; /*pct of abstinence in counselling group*/

ncouns_pct=(T1+T3)/&nocouns; /*pct of abstinence in no counselling group*/

mcont_pct=(T3+T4)/&mcont; /*pct of abstinence in multiple contest group*/

scont_pct=(T1+T2)/&scont; /*pct of abstinence in single contest group*/

/*odds ratio of counselling group*/

or_couns=((T2+T4)/(&couns-T2-T4))/((T1+T3)/(&nocouns-T1-T3));

/*odds ratio of contests group*/

or_cont=((T3+T4)/(&mcont-T3-T4))/((T1+T2)/(&scont-T1-T2));

if _NAME_="uimput1" then OR2=**1**;

if _NAME_="uimput2" then OR2=**2**;

if _NAME_="uimput3" then OR2=**3**;

if _NAME_="uimput4" then OR2=**4**;

if _NAME_="uimput5" then OR2=**5**;

if _NAME_="inf" then OR2=**999**; /*OR2=infinity*/

run;

**%mend**;

/*utemp_1 to utemp_6 contain corresponding odds ratio for counselling and contest group after urine imputation with OR1=1,2,3,4,5 and inf*/

%***urine_imput***(datain=utemp2,dataout=utemp_1,imput=imput1,f12_=f12_1,f11_=f11_1,nu=nu_1);

%***urine_imput***(datain=utemp2,dataout=utemp_2,imput=imput2,f12_=f12_2,f11_=f11_2,nu=nu_2);

%***urine_imput***(datain=utemp2,dataout=utemp_3,imput=imput3,f12_=f12_3,f11_=f11_3,nu=nu_3);

%***urine_imput***(datain=utemp2,dataout=utemp_4,imput=imput4,f12_=f12_4,f11_=f11_4,nu=nu_4);

%***urine_imput***(datain=utemp2,dataout=utemp_5,imput=imput5,f12_=f12_5,f11_=f11_5,nu=nu_5);

%***urine_imput***(datain=utemp2,dataout=utemp_6,imput=inf,f12_=f12_inf,f11_=f11_inf,nu=nu_inf);

/*Calculate the p-value based on the chisq-test using chisq_pvalue macro*/

**%macro** ***uchisq_pvalue***;

%do i=**1** %to **6**;

/*counselling group*/

%***chisq_pvalue***(datain=utemp_&i.,dataout=up_1,gr1=T2,gr2=T4,gr3=T1,gr4=T3,tot1=%str(&couns),tot2=%str(&nocouns),n_OR=**1**,OR=or2);

%***chisq_pvalue***(datain=utemp_&i.,dataout=up_2,gr1=T2,gr2=T4,gr3=T1,gr4=T3,tot1=%str(&couns),tot2=%str(&nocouns),n_OR=**2**,OR=or2);

%***chisq_pvalue***(datain=utemp_&i.,dataout=up_3,gr1=T2,gr2=T4,gr3=T1,gr4=T3,tot1=%str(&couns),tot2=%str(&nocouns),n_OR=**3**,OR=or2);

%***chisq_pvalue***(datain=utemp_&i.,dataout=up_4,gr1=T2,gr2=T4,gr3=T1,gr4=T3,tot1=%str(&couns),tot2=%str(&nocouns),n_OR=**4**,OR=or2);

%***chisq_pvalue***(datain=utemp_&i.,dataout=up_5,gr1=T2,gr2=T4,gr3=T1,gr4=T3,tot1=%str(&couns),tot2=%str(&nocouns),n_OR=**5**,OR=or2);

%***chisq_pvalue***(datain=utemp_&i.,dataout=up_inf,gr1=T2,gr2=T4,gr3=T1,gr4=T3,tot1=%str(&couns),tot2=%str(&nocouns),n_OR=**999**,OR=or2);

data ucouns_pvalue&i.;

set up_1(in=a) up_2(in=b) up_3(in=c) up_4(in=d) up_5(in=e) up_inf(in=f);

if a then OR2=**1**;

if b then OR2=**2**;

if c then OR2=**3**;

if d then OR2=**4**;

if e then OR2=**5**;

if f then OR2=**999**;

run;

/*contest group*/

%***chisq_pvalue***(datain=utemp_&i.,dataout=up_1,gr1=T3,gr2=T4,gr3=T1,gr4=T2,tot1=%str(&mcont),tot2=%str(&scont),n_OR=**1**,OR=or2);

%***chisq_pvalue***(datain=utemp_&i.,dataout=up_2,gr1=T3,gr2=T4,gr3=T1,gr4=T2,tot1=%str(&mcont),tot2=%str(&scont),n_OR=**2**,OR=or2);

%***chisq_pvalue***(datain=utemp_&i.,dataout=up_3,gr1=T3,gr2=T4,gr3=T1,gr4=T2,tot1=%str(&mcont),tot2=%str(&scont),n_OR=**3**,OR=or2);

%***chisq_pvalue***(datain=utemp_&i.,dataout=up_4,gr1=T3,gr2=T4,gr3=T1,gr4=T2,tot1=%str(&mcont),tot2=%str(&scont),n_OR=**4**,OR=or2);

%***chisq_pvalue***(datain=utemp_&i.,dataout=up_5,gr1=T3,gr2=T4,gr3=T1,gr4=T2,tot1=%str(&mcont),tot2=%str(&scont),n_OR=**5**,OR=or2);

%***chisq_pvalue***(datain=utemp_&i.,dataout=up_inf,gr1=T3,gr2=T4,gr3=T1,gr4=T2,tot1=%str(&mcont),tot2=%str(&scont),n_OR=**999**,OR=or2);

data ucont_pvalue&i.;

set up_1(in=a) up_2(in=b) up_3(in=c) up_4(in=d) up_5(in=e) up_inf(in=f);

if a then OR2=**1**;

if b then OR2=**2**;

if c then OR2=**3**;

if d then OR2=**4**;

if e then OR2=**5**;

if f then OR2=**999**;

run;

%end;

**%mend**;

%***uchisq_pvalue***;

/*Note: ucouns_pvalue1 to ucouns_pvalue6 stored p-value based on chi-square test for counselling group with OR1=1,2,3,4,5 and inf; ucons_pvalue1 to ucons_pvalue6 stored p-value based on chi-square test for contests group with OR1=1,2,3,4,5 and inf*/

********************************************************

*** Imputation and analysis for urine data ends here ***

********************************************************;

****************************************************************************

** Summarize imputed results derived above as shown in Table 3 and Table 4 **

****************************************************************************;

/*Table 3*/

**proc** **sort** data=temp3;by OR1;**run**;

**data** table3;

merge temp3 couns_pvalue(rename=(p_pchi=p_couns)) cont_pvalue(rename=(p_pchi=p_cont));

by OR1;

keep OR1 couns_pct ncouns_pct or_couns p_couns mcont_pct scont_pct or_cont p_cont;

**run**;

**data** table3;

retain OR1 couns_pct ncouns_pct or_couns p_couns mcont_pct scont_pct or_cont p_cont;

set table3;

**run**;

/*OR1=0: complete_case analysis ; OR1=999: infinity*/

**proc** **print** data=table3; title "Table 3";**run**;

/*Table 4*/

**data** ucouns_pvalue;

set ucouns_pvalue1(in=a) ucouns_pvalue2(in=b) ucouns_pvalue3(in=c) ucouns_pvalue4(in=d)

ucouns_pvalue5(in=e) ucouns_pvalue6(in=f);

if a then OR1=**1**;

if b then OR1=**2**;

if c then OR1=**3**;

if d then OR1=**4**;

if e then OR1=**5**;

if f then OR1=**999**;

**run**;

**data** ucont_pvalue;

set ucont_pvalue1(in=a) ucont_pvalue2(in=b) ucont_pvalue3(in=c) ucont_pvalue4(in=d)

ucont_pvalue5(in=e) ucont_pvalue6(in=f);

if a then OR1=**1**;

if b then OR1=**2**;

if c then OR1=**3**;

if d then OR1=**4**;

if e then OR1=**5**;

if f then OR1=**999**;

**run**;

**data** utemp3;

set utemp_1(in=a) utemp_2(in=b) utemp_3(in=c) utemp_4(in=d) utemp_5(in=e) utemp_6(in=f);

if a then OR1=**1**;

if b then OR1=**2**;

if c then OR1=**3**;

if d then OR1=**4**;

if e then OR1=**5**;

if f then OR1=**999**;

**run**;

**proc** **sort** data=utemp3;by OR1 OR2;**run**;

**data** table4;

merge utemp3 ucouns_pvalue(rename=(p_pchi=p_couns)) ucont_pvalue(rename=(p_pchi=p_cont));

by OR1 OR2;

keep OR1 OR2 couns_pct ncouns_pct or_couns p_couns mcont_pct scont_pct or_cont p_cont;

**run**;

**data** table4;

retain OR1 OR2 couns_pct ncouns_pct or_couns p_couns mcont_pct scont_pct or_cont p_cont;

set table4;

**run**;

**proc** **print** data=table4; title "Table 4"; **run**; /*OR2=999: infinity*/

**Additional file 1 Table S1.** Summary of imputation results for urine-verified abstinence assuming different levels of association between missing and abstinence when *λ* = 0.5

|  |  | Counseling vs. no counseling | | | |  | Multiple vs. single contests | | | |
| --- | --- | --- | --- | --- | --- | --- | --- | --- | --- | --- |
| ${OR}_{1}$ | ${OR}_{2}$ | Counseling  Tx2 + Tx4 | No counseling  Tx1 + Tx3 | Estimated treatment effect (odds ratio for abstinence) | P-value |  | Multiple contests  Tx3 + Tx4 | Single contest  Tx1 + Tx2 | Estimated treatment effect (odds ratio for abstinence) | P-value |
| 1 | 1 | 25.8% | 20.9% | 1.31 | .046 |  | 24.8% | 21.8% | 1.18 | .212 |
|  | 2 | 24.6% | 20.0% | 1.31 | .051 |  | 23.7% | 20.7% | 1.19 | .207 |
|  | 3 | 23.7% | 19.2% | 1.31 | .055 |  | 22.8% | 19.9% | 1.19 | .206 |
|  | 4 | 23.0% | 18.6% | 1.31 | .056 |  | 22.2% | 19.3% | 1.20 | .205 |
|  | 5 | 22.4% | 18.1% | 1.31 | .062 |  | 21.6% | 18.7% | 1.20 | .206 |
|  | +$\infty$ | 16.0% | 13.0% | 1.28 | .132 |  | 15.6% | 13.4% | 1.20 | .264 |
| 2 | 1 | 23.3% | 19.5% | 1.26 | .102 |  | 22.7% | 20.0% | 1.18 | .249 |
|  | 2 | 22.4% | 18.6% | 1.26 | .106 |  | 21.8% | 19.1% | 1.18 | .243 |
|  | 3 | 21.6% | 18.0% | 1.26 | .110 |  | 21.1% | 18.4% | 1.18 | .241 |
|  | 4 | 21.0% | 17.4% | 1.26 | .114 |  | 20.5% | 17.8% | 1.19 | .240 |
|  | 5 | 20.5% | 17.0% | 1.26 | .116 |  | 20.0% | 17.4% | 1.19 | .240 |
|  | +$\infty$ | 15.2% | 12.5% | 1.25 | .177 |  | 14.9% | 12.8% | 1.19 | .285 |
| 3 | 1 | 22.3% | 18.9% | 1.23 | .143 |  | 21.9% | 19.3% | 1.17 | .272 |
|  | 2 | 21.4% | 18.1% | 1.23 | .145 |  | 21.0% | 18.5% | 1.17 | .266 |
|  | 3 | 20.7% | 17.5% | 1.24 | .148 |  | 20.3% | 17.8% | 1.18 | .262 |
|  | 4 | 20.2% | 17.0% | 1.24 | .150 |  | 19.8% | 17.3% | 1.18 | .261 |
|  | 5 | 19.7% | 16.5% | 1.24 | .152 |  | 19.3% | 16.8% | 1.18 | .260 |
|  | +$\infty$ | 14.8% | 12.3% | 1.24 | .201 |  | 14.6% | 12.5% | 1.19 | .296 |
| 4 | 1 | 21.8% | 18.6% | 1.22 | .171 |  | 21.4% | 19.0% | 1.16 | .287 |
|  | 2 | 20.9% | 17.8% | 1.22 | .172 |  | 20.6% | 18.1% | 1.17 | .280 |
|  | 3 | 20.2% | 17.2% | 1.22 | .174 |  | 19.9% | 17.5% | 1.17 | .276 |
|  | 4 | 19.7% | 16.7% | 1.22 | .175 |  | 19.4% | 17.0% | 1.18 | .274 |
|  | 5 | 19.3% | 16.3% | 1.23 | .177 |  | 19.0% | 16.6% | 1.19 | .272 |
|  | +$\infty$ | 14.6% | 12.2% | 1.23 | .216 |  | 14.4% | 12.4% | 1.19 | .303 |
| 5 | 1 | 21.4% | 18.4% | 1.21 | .192 |  | 21.1% | 18.7% | 1.16 | .297 |
|  | 2 | 20.6% | 17.6% | 1.21 | .192 |  | 20.3% | 17.9% | 1.17 | .290 |
|  | 3 | 19.9% | 17.0% | 1.21 | .192 |  | 19.7% | 17.3% | 1.17 | .285 |
|  | 4 | 19.4% | 16.6% | 1.21 | .193 |  | 19.1% | 16.8% | 1.17 | .283 |
|  | 5 | 19.0% | 16.2% | 1.22 | .194 |  | 18.7% | 16.4% | 1.18 | .281 |
|  | +$\infty$ | 14.5% | 12.1% | 1.23 | .226 |  | 14.3% | 12.3% | 1.19 | .308 |
| +$\infty$ | 1 | 19.8% | 17.6% | 1.16 | .315 |  | 19.7% | 17.7% | 1.15 | .352 |
|  | 2 | 19.1% | 16.9% | 1.17 | .306 |  | 19.0% | 16.9% | 1.15 | .342 |
|  | 3 | 18.6% | 16.3% | 1.17 | .300 |  | 18.5% | 16.4% | 1.16 | .335 |
|  | 4 | 18.1% | 15.9% | 1.17 | .295 |  | 18.0% | 15.9% | 1.16 | .331 |
|  | 5 | 17,8% | 15.5% | 1.18 | .292 |  | 17.7% | 15.6% | 1.16 | .328 |
|  | +$\infty$ | 14.0% | 11.9% | 1.20 | .278 |  | 13.8% | 12.0% | 1.18 | .333 |

*λ*: the factor differentiating the urine missing rate between survey non-respondents and respondents (*λ* < 1 means survey non-respondents are less likely to provide urine than survey respondents); 6the case of tobacco cessation trials, t the providing behavioral (e.g. couseljects in a cigarette tobacco cessation randomize*OR*_1_: odds ratio between missing and tobacco use status for self-report data; *OR*_2_: odds ratio between urine missing and urine-verified failure among those who self-reported abstinence; Tx1: single contest + no counseling; Tx2: single contest + counseling; Tx3: multiple contests + no counseling; Tx4: multiple contests + counseling. P-values are based on the Chi-square test.
